# Achieving behaviour change for detection of Lynch syndrome using the Theoretical Domains Framework Implementation (TDFI) approach: a study protocol

**DOI:** 10.1186/s12913-016-1331-8

**Published:** 2016-03-12

**Authors:** Natalie Taylor, Janet C. Long, Deborah Debono, Rachel Williams, Elizabeth Salisbury, Sharron O’Neill, Elizabeth Eykman, Jeffrey Braithwaite, Melvin Chin

**Affiliations:** Centre for Healthcare Resilience and Implementation Science, Australian Institute of Health Innovation, Faculty of Medicine and Health, Macquarie University, Sydney, NSW 2109 Australia; Prince of Wales Hospital, Sydney, NSW Australia; Prince of Wales Clinical School, Faculty of Medicine, University of New South Wales, Sydney, Australia; NSW Pathology (SEALS), Prince of Wales Hospital, Sydney, NSW Australia; International Governance and Research Centre, Faculty of Business and Economics, Macquarie University, Sydney, Australia; NSW Pathology (SEALS), St George Hospital, Sydney, NSW Australia

**Keywords:** Implementation, Theoretical Domains Framework, Behaviour change, Hereditary cancer, Pathology

## Abstract

**Background:**

Lynch syndrome is an inherited disorder associated with a range of cancers, and found in 2–5 % of colorectal cancers. Lynch syndrome is diagnosed through a combination of significant family and clinical history and pathology. The definitive diagnostic germline test requires formal patient consent after genetic counselling. If diagnosed early, carriers of Lynch syndrome can undergo increased surveillance for cancers, which in turn can prevent late stage cancers, optimise treatment and decrease mortality for themselves and their relatives. However, over the past decade, international studies have reported that only a small proportion of individuals with suspected Lynch syndrome were referred for genetic consultation and possible genetic testing. The aim of this project is to use behaviour change theory and implementation science approaches to increase the number and speed of healthcare professional referrals of colorectal cancer patients with a high-likelihood risk of Lynch syndrome to appropriate genetic counselling services.

**Methods:**

The six-step Theoretical Domains Framework Implementation (TDFI) approach will be used at two large, metropolitan hospitals treating colorectal cancer patients. Steps are: 1) form local multidisciplinary teams to map current referral processes; 2) identify target behaviours that may lead to increased referrals using discussion supported by a retrospective audit; 3) identify barriers to those behaviours using the validated *Influences on Patient Safety Behaviours Questionnaire* and TDFI guided focus groups; 4) co-design interventions to address barriers using focus groups; 5) co-implement interventions; and 6) evaluate intervention impact. Chi square analysis will be used to test the difference in the proportion of high-likelihood risk Lynch syndrome patients being referred for genetic testing before and after intervention implementation. A paired *t*-test will be used to assess the mean time from the pathology test results to referral for high-likelihood Lynch syndrome patients pre-post intervention. Run charts will be used to continuously monitor change in referrals over time, based on scheduled monthly audits.

**Discussion:**

This project is based on a tested and refined implementation strategy (TDFI approach). Enhancing the process of identifying and referring people at high-likelihood risk of Lynch syndrome for genetic counselling will improve outcomes for patients and their relatives, and potentially save public money.

## Background

Identification and referral of patients with a relevant clinical or family history of cancer can save the lives of those affected by hereditary cancers [1]. Lynch syndrome (LS), an inherited disorder involving many types of cancer, is found in 2–5 % of colorectal cancers (CRCs) [2, 3]. Globally, there were an estimated 1.36 million new cases of CRC in 2012, with 134,349 in the United States and 40,775 in the United Kingdom [4]. In Australia there were around 17,000 new CRC cases in 2014 and this figure is projected to rise to around 20,000 by 2020 [5]. CRC (and other cancer) patients identified as being at high risk of LS can be referred for genetic counselling to Family Cancer Clinics (FCCs) where a definitive genetic test can be undertaken with the patient’s consent. This enables carriers to engage in effective screening protocols, detect and treat cancer early, and educate relatives. Early diagnosis of LS is therefore critical since surveillance (e.g., colonoscopic) and/or risk reducing surgery for LS patients and at-risk relatives can prevent cancer, optimise medical management and reduce mortality [6, 7]. Although LS has been detected in a relatively small proportion of CRC patients (5 %), tens of thousands are believed to carry a LS gene in Australia alone, making LS an extremely underdiagnosed condition [8].

Whilst clinicians cannot be expected to have detailed knowledge about causative LS genes, current guidelines from Australia, the United States and Europe emphasise their responsibility for recognising the clinical phenotype and family history characteristics of LS, and referring patients to clinical genetics or family cancer clinics if deemed necessary [9–14]. Referral is required as currently in public health, the definitive diagnostic test must be ordered by a specialised FCC and requires formal patient consent after genetic counselling. However, over the past decade, local and international studies have reported that only a small proportion of individuals with suspected LS were referred for genetic consultation and possible genetic testing [8, 15–18]. In particular, recent Australian evidence indicates that over half (54 %) of patients with CRC with a high likelihood of LS were not referred for genetic testing, despite indicators recommending referral [19]. Unidentified carriers remain unaware of their greater cancer risk or the need for ongoing screening. Relatives may also miss the opportunity to discover if they have LS. These delays in detecting and managing cancer may lead to significant morbidity and loss of life.

Implementation of clinical guidelines which aim to facilitate the translation of research evidence into practice require healthcare professionals to change their behaviour. Whilst behaviour change is complex, [20] emphasised by a growing field of research dedicated to understanding and improving healthcare, [21] it is entirely possible [22]. The application of behaviour change methods to design interventions, such as identifying and addressing key barriers to change, [23] can transform healthcare organisations and improve patient outcomes [23, 24]. Whilst some evidence exists for the factors affecting clinical decisions and actions for patients who may benefit from screening to detect LS, [10] the development and implementation of evidence-based interventions to address health care professionals’ barriers to referring patients with CRC with a high-likelihood of LS for genetic testing is lacking. Using approaches such as intervention co-design with key stakeholders can enhance intervention generalisability across different contexts, and the translation of effective approaches from research into practice [25].

The validated, six-step Theoretical Domains Framework Implementation (TDFI) approach [26] uses behaviour change theory and implementation science principles to identify and address key barriers to changing clinical practice. Barriers are represented by 11 domains of behaviour change, e.g., ‘knowledge’, ‘skills’, ‘social influences’, ‘emotion’, ‘environment’, ‘professional role and identity’, ‘memory, attention, decision processes’. These domains are based on theoretical constructs from multiple psychological and organisational behaviour change theories [24]. Authenticating a bottom-up strategy with senior management support, the TDFI approach takes a team of front-line health care professionals supported by behavioural scientists through a collaborative implementation process. We have successfully used this approach to demonstrate clinical, statistical and cost-effective improvements in guideline implementation (e.g., for anaesthetics, enteral feeding) across UK hospitals [20, 22, 26]. In this project we will use the validated TDFI approach in two large Australian hospitals to improve the timely identification of LS in patients with CRC. This represents an opportunity to address a known clinical problem and unmet need through the application of a behavioural change approach.

## Methods/Design

### Overview

**Fig. 1:**
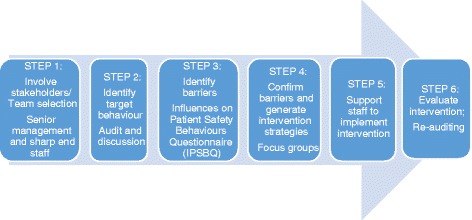
The Theoretical Domains Framework Implementation Approach [26]

We will use the TDFI approach in two large Australian metropolitan hospitals to: 1) form health care professional implementation teams and process map LS referrals; 2) conduct baseline audits of CRC surgery patients and LS genetic testing referrals to identify target behaviours for change; 3) use the validated *Influences on Patient Safety Behaviours Questionnaire* (IPSBQ) [20] and undertake TDF-guided focus groups with health care professionals to identify and verify referral barriers (e.g., knowledge, environment/resources, memory, emotion); 4) co-design interventions with health care professionals using evidence-based strategies to address key barriers; 5) co-implement interventions; and 6) evaluate effectiveness using audit and questionnaire data to assess practice and culture change (see Fig. 1).

### Recruitment

#### Hospital recruitment (preliminary work)

The project team secured commitment in principle from two large metropolitan Australian hospitals and associated healthcare organisations involved in the LS referral process (i.e., hospitals, Family Cancer Clinics, and pathology laboratories) whilst forming the project plan as part of a translational research funding application. This was achieved by liaising with key stakeholders (i.e., hospital oncologists, pathologists, and genetics counsellors) to identify if and how the context-specific problem could be addressed through this behaviour change implementation approach. Upon receiving notification of the funding award, the project team have confirmed hospital participation through invitations to participate and via ethics and site-specific governance procedures, which require authorising signatures from senior management at each organisation.

#### Staff

The process of recruiting staff to the implementation teams is described in step 1 of the procedure. Recruitment of health care professionals for self-report psychosocial measures of perceived barriers to behaviour change will use implementation team organisational networks, as described in Taylor et al. [22, 26]. All staff for whom the key behaviour is relevant (e.g., colorectal surgeons and medical oncologists, pathologists, and cancer nurses) will be targeted.

### Procedure

#### Step 1 – Implementation team selection

We will identify, through referral and expression of interest, up to 10 multidisciplinary change agents [27] at each of the hospitals to form an implementation team that will work with investigators. The change agents will assist in: mapping and critiquing the current process; gathering audit data; introducing the project to the various components of the referral process; and identifying possible target behaviour(s) for change (e.g., methods of reporting or disseminating pathology results, use of guidelines to inform referral decisions, referral methods).

#### Step 2 – Audit of current practice

We will audit the following data to identify baseline evidence of current referral processes: 1) number and date of colectomy samples received from patients with CRC by each local pathology service; 2) number and date of mismatch repair (MMR) deficient tumours detected through immunohistochemistry (IHC) which are associated with LS, identified in these resected specimens; 3) number and date of referrals to the FCC for genetic counselling about testing for LS; 4a) number of patients who chose to take a genetic test, and of those patients, 4b) number diagnosed with LS, and number not diagnosed with LS (including dates of results). Details on the wording of the pathology report received by the treating health care professionals such as whether LS is mentioned and any recommendations for referral to the FCC will be noted. This information will allow the team to identify the proportion of patients with CRC with high-likelihood risk of LS, compared to the number of referrals made for genetic testing; i.e., the accuracy of referral practices (see Table 1), as well as the speed of referrals, and define the target behaviour(s) for change. This data will also be used to identify post-intervention implementation changes in referral practices and speeds.

**Table 1:** Matrix demonstrating the information that data collection will provide regarding accuracy of LS referral practices

LS risk status	Referred	Not referred
Patients with CRC with high likelihood of LS	✓	✗
Patients with CRC with low likelihood of LS	✗	✓

#### Step 3 – Identification of barriers to change

The validated IPSBQ [20] will be distributed to approximately 25 health care professionals involved in the LS referral process (e.g., CRC surgeons, oncologists, pathology laboratory staff, FCCs) in each hospital, in both paper and online format. The IPSBQ will be used to assess the barriers (e.g., knowledge, environment/resources, memory, emotion) to performing the target behaviour(s) according to the domains of the TDF. Following analysis of the questionnaire data, stage 1 of TDFI-guided focus groups will be undertaken with healthcare professionals to discuss key barriers to clinical practice change in detail.

#### Step 4 – Development of solutions to overcome barriers

Stage 2 of the TDFI-guided focus groups will consist of project team members working with staff to devise context-relevant interventions to overcome key barriers using their expertise in evidence-based behaviour change strategies (e.g. [28, 29]) and implementation science (e.g. [30, 31]) methods. This development phase will involve consultation with our consumer networks to gain a public perspective on the intended intervention content and function.

#### Step 5 – Implementation of solutions

A report outlining the process, findings, and suggested interventions will be submitted to senior management in each hospital. Following authorisation for implementation, the project team, with support from the implementation team, will implement intervention strategies.

#### Step 6 – Evaluation

Monthly collection of pathology and referral data will be undertaken to assess the impact of interventions and to allow for refinements. Pre-post intervention data analysis will be undertaken by the project team to determine the impact of the intervention on the proportion of high-likelihood LS risk CRC patient referrals for LS testing, and speed of referrals. Post-intervention implementation the IPSBQ will be used to assess changes in perceived healthcare professional barriers to referring high-likelihood risk of LS patients for genetic testing.

### Measures

#### Access to patient data and data collection staff

The heads of pathology for each participating site will facilitate access to retrospective and prospective pathology data for all the MMR deficient tumours identified through routine IHC testing. A senior genetic counsellor (RW) will facilitate access to referral data in the FCC database. A pathology registrar (EE) with clinical authorisation to access hospital pathology data will be responsible for undertaking this aspect of the data collection.

#### Identifying the proportion of high-likelihood risk patient referrals

MMR deficient tumour data will be cross-referenced against the number of CRC patient LS referrals using the FCC database. This will identify the proportion of high-likelihood risk of LS cases (denominator) compared to the number of referrals made for genetic testing (numerator).

#### Identifying the speed of referrals

Pre-post intervention implementation samples of high-likelihood risk of LS patients who were referred for testing will also be identified, and (where available) the calendar dates of events occurring within the referral process (e.g., tumor resection, identification of MMR deficient tumours, referral recommendation, date referral received by genetics to discuss genetic testing) will be recorded. This will allow us to determine the change in the speed of referral for genetic testing from the initial recognition of pathology test results.

#### Identifying health care professionals’ perceptions of barriers to referral

The validated IPSBQ [20] will be distributed to relevant health care professionals pre-post implementation through steering group members. This will allow us to identify change in the perceptions of barriers to referral.

### Analysis plan

#### Proportion of high-likelihood LS risk patient referrals

We will use Chi square to test: a) the difference in the proportion of high-likelihood LS patients being referred for genetic testing before and after intervention implementation, and b) to detect changes in the sensitivity of the system [i.e., for the number of referrals of individuals with the genetic mutation (true positive), compared with the number of referrals made for individuals who do not have the genetic mutation (false positive)]. Throughout the course of the project, we will use run charts to continuously monitor change in referrals over time, based on the scheduled monthly audits. This will provide insights into which aspects of the implemented interventions are effective.

#### Speed of referrals

A paired *t*-test will be used to assess the mean time from the pathology test results to referral for high-likelihood LS patients before and after the intervention. Where possible, we will also assess for effects of moderators on overall outcomes, such as where pathology test results are sent, or by which type of clinician a referral is made.

#### Health care professional perceptions of barriers to referral

Inter-item correlations will be used to test for internal consistency of each subscale of the IPSBQ, with values above 0.15 to 0.50 being the optimal range. Criterion validity will be established by comparing mean total questionnaire scores against audited referral rates in each organisation. Multivariate analysis of variance (MANOVA) will be used to assess the difference in perceived barriers post-intervention implementation, after controlling for baseline differences.

### Trial status

Data collection for this study is ongoing. Of the six-step process described above, Steps 1–3 (recruitment of implementation teams, generation of a process map, retrospective audit, and collection of the IPSBQ questionnaire) have been completed. Step 4 is underway.

## Discussion

This project is based on a refined, tested collaborative implementation strategy that will be applied to identify and overcome key healthcare professional barriers to appropriately referring patients with a high-likelihood risk of hereditary cancer genes for genetic testing. It will introduce regular monitoring of LS referral practices to participating organisations, thereby increasing awareness of LS and the potential for sustained, improved referral practice. By applying and refining the tested TDFI approach, for the first time within the Australian healthcare system and with healthcare professionals to improve cancer care, we expect to improve the current rates of referral for genetic testing in New South Wales (NSW) from 46 % [19] to ensure those patients with CRC with a high likelihood of LS are rapidly identified and provided with the opportunity to have the definitive genetic test. This is expected to lead to better cancer prevention through the provision of opportunities for more frequent targeted screening, and access to surgical procedures to remove tissues and organs at higher risk of LS related cancers, [6] resulting in improved patient outcomes, including gains in years of life [32].

In addition to improved patient outcomes, by increasing the number of high-likelihood LS referrals for genetic testing, the TDFI approach may also save public money. For example, tumour testing for LS (on which referrals are based, alongside other factors such as family history) is cost effective in the UK, with all incremental cost-effectiveness ratios below the National Institute for Health and Care Excellence threshold of <£20 K(Aus$36 K) per quality adjusted life year (QALY) gained [32]. Other potential outcomes might include: improved service coordination, more effective organisational functioning, improved inter-professional collaboration and communication between health care professionals, improved health care professional job satisfaction with improved patient outcomes, reductions in crisis work with late detection and removal of life threatening cancers. Overall the outcomes of each of these advances will serve to benefit patients through improved evidence-based surveillance and faster treatment.

To disseminate the findings of the project we will develop a strategy for effectively sharing lessons and resources within and between participating organisations, and with other healthcare organisations in Australia and internationally. This might involve, for example, liaising with key local, national, and international cancer networks (e.g., translational research networks, cancer agencies including the Cancer Institute New South Wales and consumer agencies such as Lynch Syndrome Australia) to devise ways for communicating the outcomes of this work into community based and academic settings. These activities will enable interventions developed and lessons learned through this work to be shared with healthcare organisations and the wider community both within and beyond the participating hospital networks to improve referral patterns of patients with CRC with high-likelihood risk of LS.

### Ethics approval and consent to participate

Ethical approval and site specific governance has been granted for this study by the South Eastern Sydney Local Health District Human Research Ethics Committee (reference: 15/103). Informed written consent has been obtained from all study participants recruited to date, and informed verbal or written consent will be obtained from all future study participants.

## Abbreviations

CRC: colorectal cancer
FCC: Family Cancer Clinic
IHC: immunohistochemistry
IPSBQ: Influences on Patient Safety Behaviours Questionnaire
LS: Lynch syndrome
MMR: Mismatch Repair
NSW: New South Wales
TDFI: Theoretical Domains Framework Implementation
